# MCT1 as a critical regulator of insulin signaling, energy homeostasis and podocyte function

**DOI:** 10.1038/s41598-026-37093-x

**Published:** 2026-01-21

**Authors:** Maria Szrejder, Irena Audzeyenka, Patrycja Rachubik, Dorota Rogacka, Agnieszka Piwkowska

**Affiliations:** Mossakowski Medical Research Institute, Laboratory of Molecular and Cellular Nephrology, Polish Academy of Sciences, Wita Stwosza St. 63, Gdańsk, 80–308 Poland

**Keywords:** MCT1, Lactate metabolism, Glomerular permeability, Insulin signaling, Cell signalling, Cytoskeleton

## Abstract

Podocytes are highly specialized epithelial cells that play a central role in maintaining integrity of the glomerular filtration barrier. Because of their complex architecture and dynamic actin-based cytoskeleton, podocytes have substantial energy requirements, which are predominantly supported by glycolysis. Insulin signaling and glucose uptake are key regulators of cytoskeletal dynamics in these cells. Recent evidence highlights the importance of lactate metabolism in maintaining podocyte metabolic homeostasis, supported by a well-developed system for controlling lactate levels. Monocarboxylate transporter 1 (MCT1), a principal mediator of lactate transport, has emerged as a critical regulator of cellular energy balance. The present study investigated the role of MCT1 in insulin-stimulated glucose metabolism and its impact on podocyte morphology and function. Our findings showed that MCT1 inhibition impaired glucose uptake and suppressed glycolytic flux. This metabolic disruption was accompanied by alterations of the localization of key insulin signaling proteins, disorganization of the actin cytoskeleton, and an increase in permeability of the podocyte layer. Interestingly, MCT1 inhibition also triggered a compensatory shift toward oxidative phosphorylation, potentially linked to an increase in mitochondrial biogenesis. These results underscore the pivotal role of MCT1 in regulating glucose metabolism and actin cytoskeleton organization in podocytes and suggest that lactate transport is essential for preserving their structure and function. Targeting MCT1 and lactate metabolism may offer a novel therapeutic strategy for glomerular diseases that are characterized by insulin resistance and metabolic dysregulation.

**Supplementary Information:**

The online version contains supplementary material available at 10.1038/s41598-026-37093-x.

## Introduction

Podocytes are highly specialized epithelial cells that form a crucial component of the glomerular filtration barrier, specifically the slit diaphragm. This intricate structure consists of interdigitating foot processes, supported by a dynamic actin-based cytoskeleton and contractile machinery that enable rapid shape remodeling in response to filtration demands^1,2^. Because of their complex architecture, podocytes have significant metabolic requirements to precisely regulate their function^2–4^. Recent studies showed that the dysfunction of energy transduction within podocytes may contribute to injury in various glomerular diseases^4–8^.

Podocytes rely on multiple metabolic pathways to generate energy, with glucose metabolism playing a central role^9–11^. They are uniquely insulin-sensitive cells, in which insulin stimulation drives glucose uptake through glucose transporter 4 (GLUT4) translocation^11,12^. Insulin binds to its receptor (IR), activating the PI3K/Akt pathway, which in turn promotes GLUT4 translocation to the plasma membrane and facilitates glucose uptake^13^. Pyruvate, the primary product of glycolysis, can either enter mitochondria for oxidation via the tricarboxylic acid cycle or be reduced to lactate. The interconversion between pyruvate and lactate is regulated by lactate dehydrogenase (LDH) activity and the cellular availability of nicotinamide adenine dinucleotide (NAD⁺)^15^. This dynamic interplay is central to the lactate shuttle hypothesis, which posits that lactate is a central regulator of redox balance and energy homeostasis^15^. Lactate acts both as a crucial metabolic substrate that supports oxidative metabolism and gluconeogenesis and as a signaling molecule that facilitates coordinated communication across cells, organs, and tissues. Recent research has highlighted the essential role of lactate in podocyte metabolism, underscoring its significance in maintaining structure and function of the glomerular filtration barrier, which depends on a steady energy supply^10,16,17^,. Our recent findings demonstrated that podocytes are capable of utilizing lactate as an alternative energy source^16^. Under glucose deprivation conditions, supplementation with L-lactate preserved cell survival, sustained glycolytic activity, and prevented glycogen reserve depletion^16^.

Lactate flux across the plasma membrane occurs through a facilitated diffusion mechanism, functioning as a lactate-proton cotransport system. Proton-linked monocarboxylate transporters 1–4 (MCT1-4) facilitate the movement of monocarboxylates, such as lactate, pyruvate, and ketone bodies, each with distinct tissue distributions^18^. MCT1 is ubiquitously expressed and supports the influx of these substrates, depending on metabolic conditions, whereas MCT4 predominantly facilitates lactic acid efflux^19^. We recently reported the presence of lactate transporters in primary rat podocytes^16^. Furthermore, the observed changes in transporter levels in response to limited glucose availability and L-lactate supplementation suggest that podocytes can rapidly adapt to metabolic fluctuations, further supporting lactate’s pivotal role in their metabolic flexibility.

The remarkable energy adaptability of podocytes is demonstrated by their ability to generate adenosine triphosphate (ATP) through both glycolysis and oxidative phosphorylation (OXPHOS)^10^. Abe et al. reported that the inhibition of either glycolysis or OXPHOS led to a reduction of ATP levels, highlighting the importance of both pathways^10^. However, evidence indicates that podocytes exhibit a predominantly glycolytic phenotype^21^, with glucose fermentation to lactate serving as a key energy source^17^. Similar metabolic dependence was demonstrated in our previous studies, in which the presence of extracellular lactate was associated with lower glucose uptake, the downregulation of GLUT4 expression, and a consequent decrease in glycolytic capacity^16^ with a metabolic shift toward OXPHOS^21^. This shift was further supported by an increase in intracellular pyruvate levels and an increase in LDH activity. Additionally, the mitochondrial localization of both LDH isoforms (LDHA and LDHB), along with an increase in the mitochondrial expression of MCT1 and MCT2, was observed under lactate supplementation conditions^21^. Collectively, these findings support the concept that podocytes preferentially utilize lactate over glucose, transporting it into mitochondria for further oxidation via the tricarboxylic acid cycle.

Whole-body lactate turnover was shown to play a significant role in the development of insulin resistance^17,22,23^. The insulin signaling cascade and GLUT4 trafficking are tightly connected to organization of the F-actin cytoskeleton, which plays a crucial role in maintaining podocyte structure and function^25^. Impairments in insulin signaling in podocytes disrupt the cytoskeleton, leading to foot process flattening and detachment from the glomerular basement membrane^26^. These structural changes result in irreversible podocyte loss and proteinuria, a hallmark of kidney damage^26^. The dysregulation of energy metabolism has been implicated in podocyte injury, driving structural alterations and the effacement of foot processes^27^. Thus, the present study investigated the impact of blocking MCT1 on insulin-stimulated glucose metabolism and the morphology and function of podocytes.

## Materials and methods

### Preparation and culture of primary rat podocytes

The study was reported in accordance with ARRIVE guidelines. All of the experimental procedures were performed in accordance with directive 2010/63/EU and were approved by the local Bioethics Commission in Bydgoszcz. We used primary rat podocytes isolated from Wistar rats obtained from the Animal Facility of the Mossakowski Medical Research Institute, as described previously^28^. The experiments were conducted using podocytes that were cultivated for 12–20 days. Cell phenotypes were established using podocyte-specific antibodies against Wilms tumor-1 protein (Biotrend, Koeln, Germany) and synaptopodin (Progen, Heidelberg, Germany). Podocytes were cultured in standard medium that contained 11.1 mM glucose (standard glucose). On the day of the experiment, cells were preincubated for a of 30 min with α-cyano-4-hydroxycinnamic acid (CHC; 1 mM), a non-selective monocarboxylate transporter (MCT) inhibitor with the highest affinity for MCT1²⁸. They were then stimulated with either bovine insulin (300 nM, 5 min) or lactate (10 mM, 30 min).

### Glucose uptake assay

Glucose uptake was measured as described previously with minor modifications^29^. Cells were washed three times with warmed Ca^2+^ and Mg^2+^-free phosphate-buffered saline (PBS) and incubated for 2 h with serum- and glucose-free RPMI 1640 medium with the addition of CHC (1 mM, 30 min). Glucose uptake was measured using 0.5 µCi/well of (1,2^3^H)- deoxy-D-glucose diluted in non-radioactive glucose at a final concentration of 50 µM with or without 300 nM insulin for 5 min. Radioactivity was measured by liquid scintillation counting using a MicroBeta2 Microplate Counter (Perkin Elmer).

### Western blot

The protocols for cell lysate preparations and Western blot analyses were described previously^28^. Primary antibodies are listed in Table 1. The densitometric analyses were performed using Quantity One software (Bio-Rad, Hercules, CA, USA).

**Table 1:** Primary antibodies used in the experiments.

Antigen	Clonality	Dilution	Source	Catalog no.
MCT1	polyclonal	1:1000	Merck Millipore	AB1286-I
IRβ	monoclonal	1:200	Santa Cruz Biotechnology	sc-81,465
p-IRβ	monoclonal	1:200	Santa Cruz Biotechnology	sc-80,500
Akt1/2/3	monoclonal	1:200	Santa Cruz Biotechnology	sc-81,434
p-Akt1/2/3	monoclonal	1:200	Santa Cruz Biotechnology	sc-81,433
β-actin	monoclonal	1:20000	Sigma-Aldrich	A5441

### Immunofluorescent staining

Podocytes were seeded on type I collagen-coated coverslips (Becton Dickinson Labware, Beckton, UK) and cultured in standard glucose medium. The cells were then preserved in 4% paraformaldehyde in PBS for 20 min at room temperature and permeabilized in 0.3% Triton X-100 for 2 min. The cells were then blocked in blocking buffer (PBS that contained 2% fetal bovine serum, 2% bovine serum albumin, and 0.2% fish gelatin) for 1 h. After blocking, the cells were incubated overnight with the following primary antibodies at 4 °C: anti-GLUT4 (Santa Cruz Biotechnology, catalog no. sc-53566, 1:50), anti-MCT1 (Abclonal, catalog no. A3013, 1:50), and anti-nephrin (Sigma Aldrich, catalog no. PRS2265, 1:200).

Next, the cells were washed three times with cold PBS and incubated with secondary antibodies conjugated with Alexa Fluor 488 (1:200) or Alexa Fluor 546 (1:200) at 4 °C for 60 min. Actin was stained with Alexa Fluor 488 phalloidin (1:200, catalog no. A12379, Thermo Fisher Scientific). Specimens were imaged using a confocal laser scanning microscope and NIS-Elements software (Nikon) with a 63× oil immersion lens.

### Adenosine triphosphate rate assay

A Seahorse Bioscience XFp Extracellular Flux Analyzer was used to measure the oxygen consumption rate (OCR) and extracellular acidification rate (ECAR) in podocyte cell cultures. Cells were seeded and differentiated on eight-well culture microplates (Agilent). On the day of the experiment, the growth medium was replaced with assay medium (XF minimal Dulbecco’s Modified Eagle Medium supplemented with 10 mM D-glucose, 1 mM sodium pyruvate, and 2 mM L-glutamine) for 1 h at 37 °C without CO_2_. The OCR values were determined before and after an injection of the MCT1 inhibitor CHC (1 mM), insulin (300 nM) or lactate (10 mM), oligomycin (1 µM), and rotenone + antimycin A (0.5 µM). Adenosine triphosphate rates were obtained from slopes of OCRs and ECARs in real-time analyses. The results were normalized to protein concentrations that were determined for each cell culture plate well using the Bradford method. Based on the measurements, we estimated the relative contribution of glycolysis and mitochondrial respiration to total ATP production.

### Transepithelial permeability assay

Transepithelial permeability to albumin was evaluated by measuring the diffusion of FITC-labeled bovine serum albumin (BSA) across the podocyte monolayer as described previously^28^. Briefly, for this experiment, podocytes were first cultured on cell culture inserts that were coated with type IV collagen. Before the experiments, the medium was replaced with serum-free RPMI1640 (SFM) for 2 h. Afterward, the medium in the upper chamber was replaced with fresh SFM, while SFM was added in the lower chamber with fluorescently labeled (FITC) BSA at a concentration of 1 mg/ml. After 1 h, the solution from the upper compartment was transferred to a 96-well plate, and the FITC-albumin concentration was determined based on reading absorbance at 490 nm in a plate spectrophotometer (ELx808, BioTek).

### Isolation of glomeruli

The protocol for the isolation of glomeruli was performed as described previously^30^. Briefly, the kidneys were excised and placed in ice-cold PBS (pH 7.4) that was supplemented with 0.49 mM MgCl2, 0.9 mM CaCl2, and 5.6 mM glucose. The renal capsule was removed, and the cortex was minced and pressed through a series of sieves with decreasing pore diameters (250, 125, and 75 μm). The resulting suspension contained decapsulated glomeruli. The entire procedure was carried out on ice and completed in under 1 h.

### Glomerular permeability to albumin in vitro

The volume response of glomerular capillaries to an oncotic gradient that is generated by changing concentrations of albumin was analyzed as described previously^31^. The volume response of glomerular capillaries to an oncotic gradient, created using defined concentrations of albumin, was measured. Briefly, isolated glomeruli were fixed on poly-L-lysine-coated glass coverslips and incubated in a medium that contained 5% BSA and lactate in the presence or without CHC (1mM, 20 min) at 37 ◦C. The glomeruli were then washed with 5% BSA medium to remove the lactate and CHC. Finally, the glomeruli were placed in a medium that contained 1% BSA to generate an oncotic gradient across the capillary wall, while control glomeruli were incubated in fresh 5% BSA medium (without the oncotic gradient). Changes in glomerular volume (V) were recorded by videomicroscopy (Olympus IX51) and calculated according to the following formula: V = [4/3A√ A/π]/106, where A is the glomerular surface area (calculated with CellSens Dimension 1.18 software, Olympus, Hamburg, Germany). Volume changes (∆V) were calculated using the formula ∆V = (Vfinal − Vinitial)/Vinitial, where the increase in ∆V is directly related to the oncotic gradient applied across the capillary wall. This principle was used to calculate the reflection coefficient of albumin (σalb), defined as the ratio of ∆V for experimental glomeruli to ∆V of control glomeruli in response to identical oncotic gradients, where σalb = ∆Vexperimental/∆Vcontrol. The reflection coefficient of albumin (convectional Palb) was used to calculate glomerular capillary permeability to albumin (1 − σalb), which describes the movement of albumin consequent to water flow.

### Statistical analysis

All statistical analyses were performed using GraphPad Prism 8 software. The Shapiro-Wilk test was used to determine whether parametric or nonparametric tests should be conducted. The statistical analyses were performed using one-way analysis of variance. The data are expressed as the mean ± SEM. Values of *p* < 0.05 were considered statistically significant.

## Results

### Effect of MCT1 Inhibition on insulin-stimulated glucose uptake

**Fig. 1:**
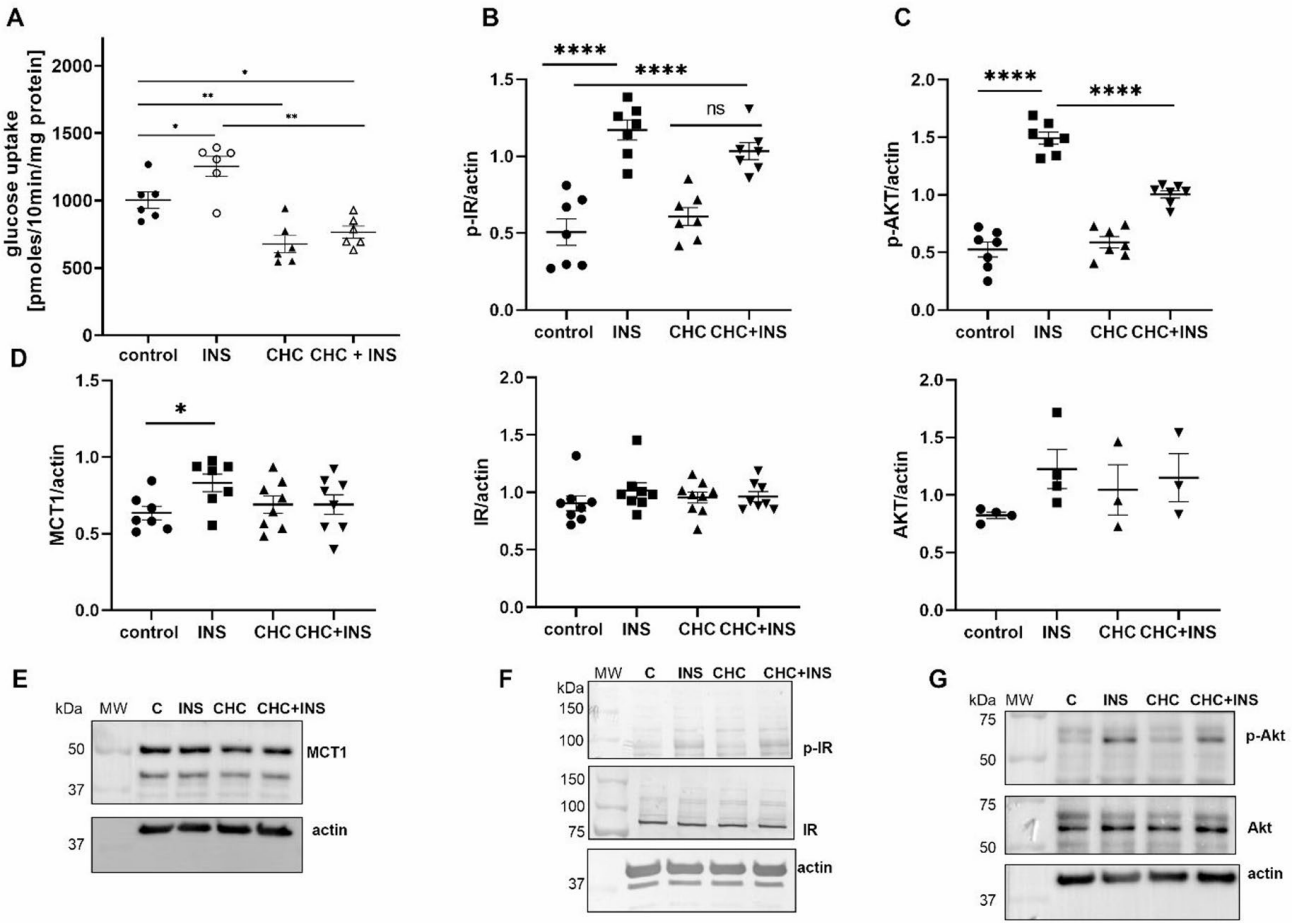
Effect of MCT1 inhibition on insulin-stimulated glucose uptake and insulin signaling in podocytes. (**A**) Basal and insulin (INS)-dependent glucose uptake in podocytes. Glucose uptake measurements began with the addition of 0.5 µCi of (1,2^3^H)- deoxy-D-glucose diluted in non-radioactive glucose to a final concentration of 50 µM and 300 nM INS. Glucose uptake was measured for 5 min. The results are expressed as the mean ± SEM (*n* = 6). *p < 0.05, **p < 0.01, compared with control; **p < 0.01, compared with INS. (**B-D**) Cell lysates were analyzed by immunoblotting using anti-pIR, anti-IR, anti-pAKT, anti-AKT, anti-MCT1, and anti-β-actin antibodies. The data are expressed as the mean ± SEM (*n* = 3–9). *p < 0.05, ****p < 0.0001, compared with control; ****p < 0.0001, compared with INS. (**E-G**) Representative immunoblots of the quantitative densitometric analysis.

**Fig. 2:**
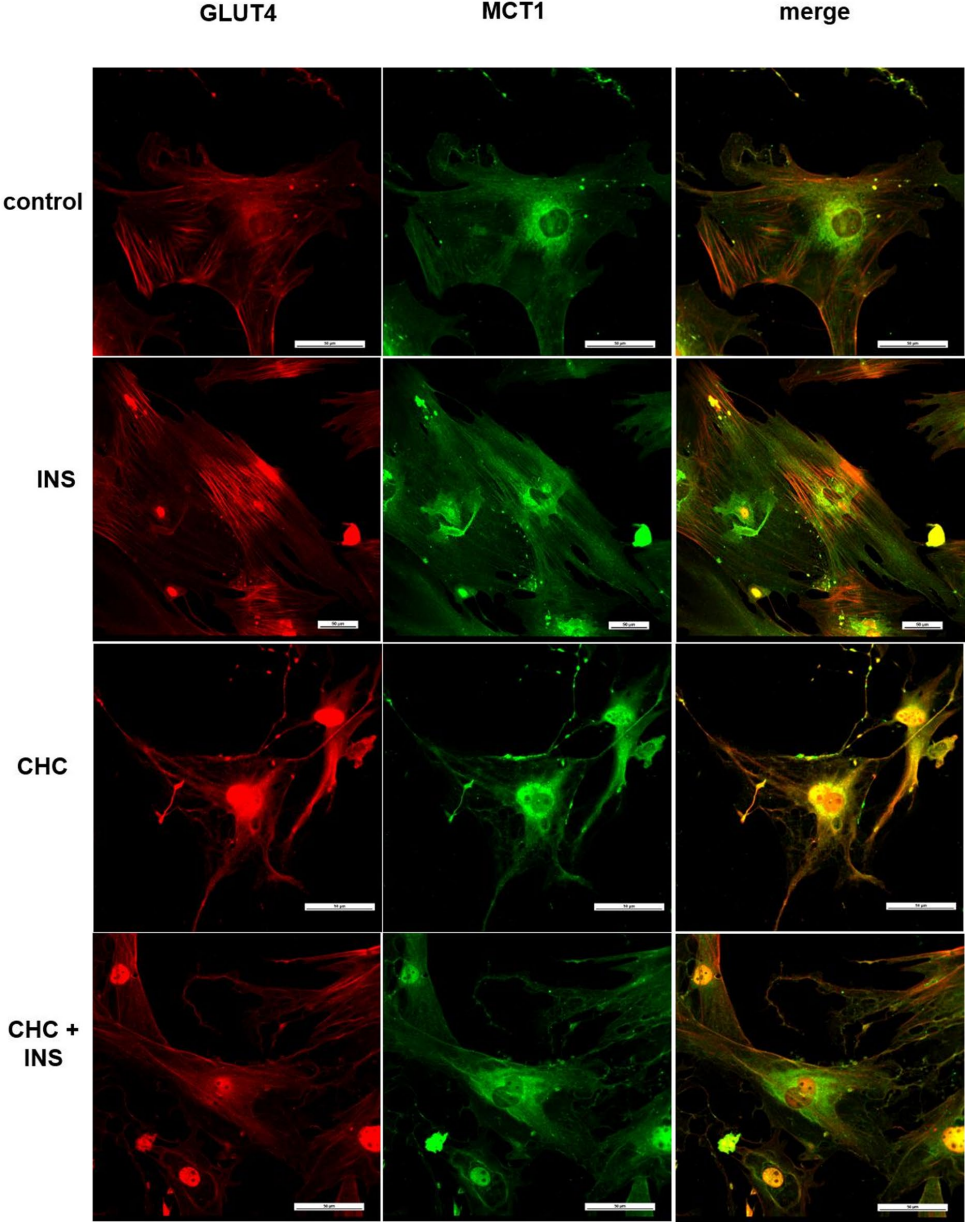
Effect of MCT1 inhibition on the cellular distribution of GLUT4 and MCT1 proteins in podocytes. Representative images are shown of GLUT4 and MCT1 localization within podocytes. Cells were immunostained with anti-GLUT4 or anti-MCT1 antibodies.

In a previous study, we found that exposing podocytes to extracellular lactate led to reductions of both glucose uptake and GLUT4 expression^16^. Among the MCTs, MCT1, which is encoded by *Slc16a1*, is the most well-characterized and plays a key role in the uptake of L-lactate for oxidative metabolism^32,33^. Therefore, in the present study, we investigated the effect of MCT1 inhibition on glucose uptake in rat podocytes. Although CHC is a non-selective inhibitor of MCTs, previous studies have shown that it exhibits relatively higher selectivity for MCT1 compared to other isoforms and is therefore commonly used as a functional MCT1 inhibitor^34–37^. Our results demonstrated that inhibition of MCT1 with CHC significantly reduced glucose uptake under basal conditions (32%, *p* = 0.0014; Fig. 1A) and prevented its increase in response to insulin (39%, *p* = 0.0037; Fig. 1A). Similarly, blocking MCT1 inhibited the insulin-induced phosphorylation of AKT protein by ~ 33% (from 1.49 ± 0.05 to 1.00 ± 0.03, *p* < 0.0001; Fig. 1C, G), a key downstream effector in the insulin signaling pathway that is responsible for mediating glucose uptake. However, MCT1 inhibition did not alter insulin-induced insulin receptor phosphorylation (Fig. 1B, F), suggesting that its effect on insulin signaling occurs downstream of the insulin receptor. Immunofluorescence experiments revealed an increase in GLUT4 staining near the cell surface after insulin treatment, whereas CHC alone reduced this signal. Notably, preincubation with the MCT1 inhibitor CHC appeared to prevent insulin-induced changes in GLUT4 localization (Fig. 2). Interestingly, insulin treatment increased MCT1 protein levels by ~ 31% (*p* = 0.0261), an effect that was absent under MCT1-inhibiting conditions (Fig. 1D-E). Similarly, we observed an intensification of MCT1 immunofluorescent staining after insulin treatment, which was diminished after CHC administration (Fig. 2). Additionally, insulin stimulation led to a more cytoplasmic distribution of MCT1, while CHC treatment promoted its perinuclear localization (Fig. 2.). Under the combined conditions, insulin appeared to counteract the effect of CHC, in which MCT1 localization resembled localization that was observed under control conditions.

### Effect of MCT1 Inhibition on ATP production rate in the presence of insulin and lactate

**Fig. 3:**
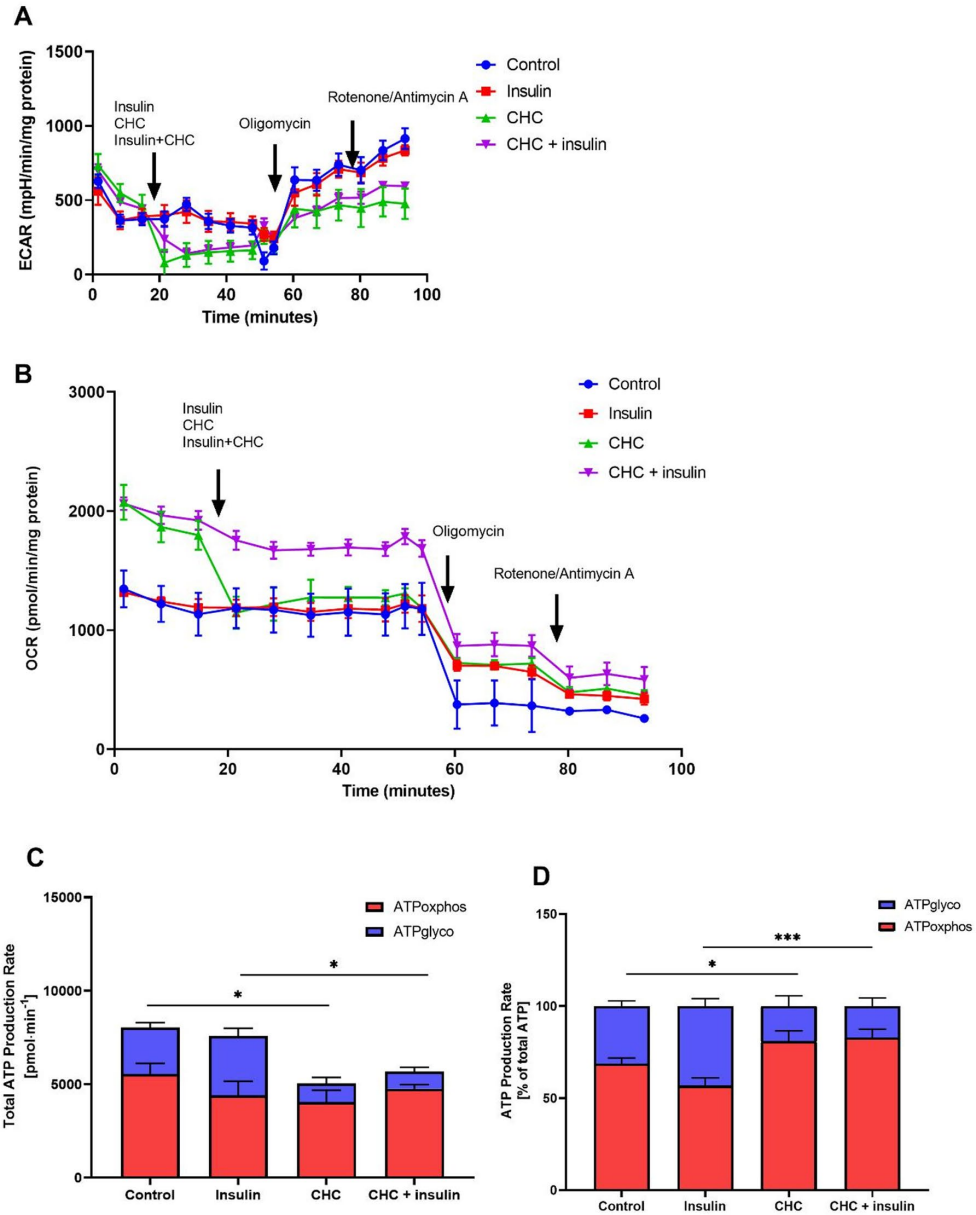
Influence of MCT1 inhibition on ATP production in the presence of insulin. Using the Seahorse XFp Extracellular Flux Analyzer, measurements of the extracellular acidification rate (ECAR) (**A**) and oxygen consumption rate (OCR) (**B**) were performed in podocytes to assess the relative contributions of glycolysis and mitochondrial respiration to total ATP production (**C**,** D**). Basal respiration was first recorded, after which compounds were sequentially injected: CHC (1mM), insulin (300nM), oligomycin (1 µM) and rotenone/antimycin A (0.5 µM). The data are expressed as the mean ± SEM (*n* = 4–6). *p < 0.05, compared with control; *p < 0.05 ***p < 0.001, compared with insulin.

**Fig. 4:**
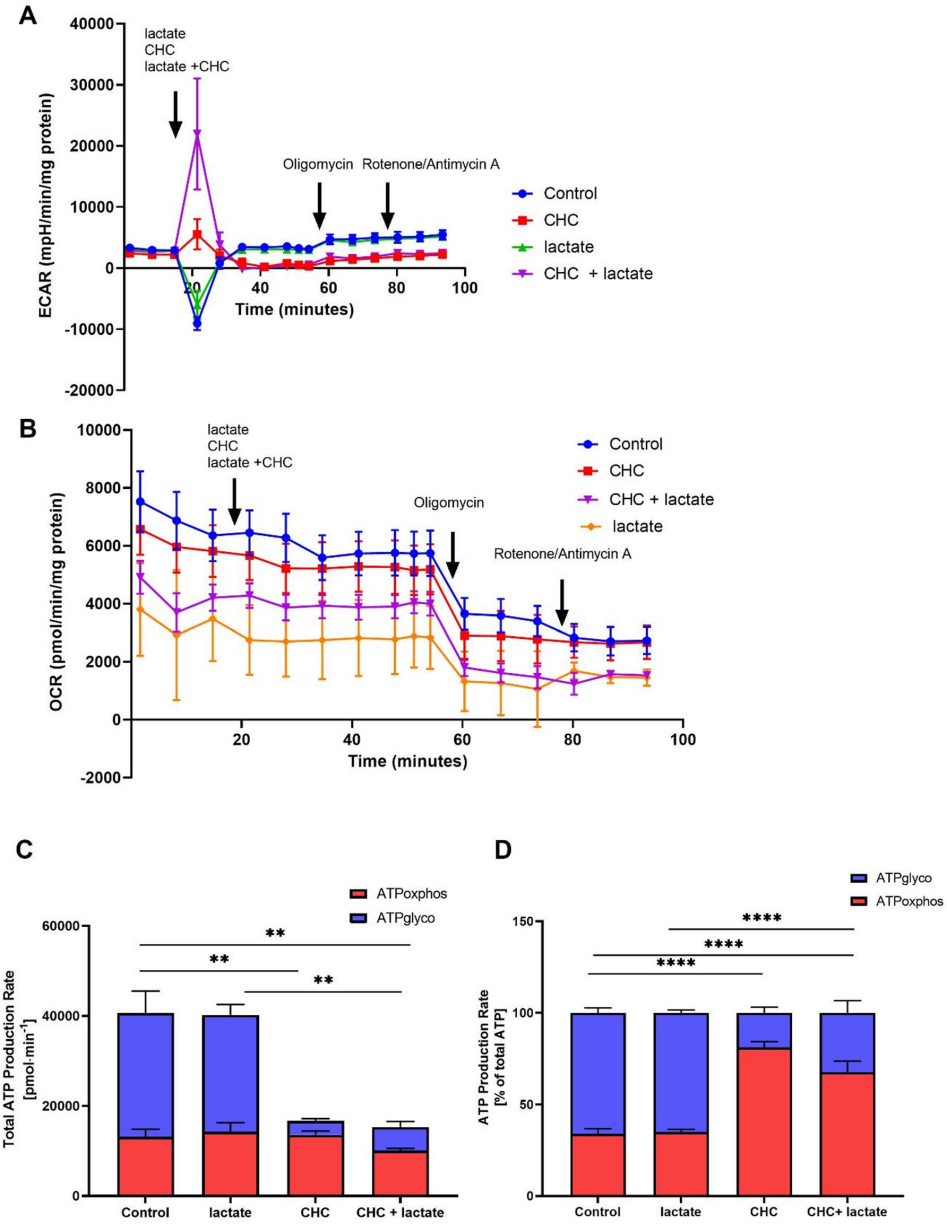
Influence of MCT1 inhibition on ATP production in the presence of lactate. Using the Seahorse XFp Extracellular Flux Analyzer, measurements of the extracellular acidification rate (ECAR) (**A**) and oxygen consumption rate (OCR) (**B**) were performed in podocytes to assess the relative contributions of glycolysis and mitochondrial respiration to total ATP production (**C**,** D**). Basal respiration was first recorded, after which compounds were sequentially injected: CHC (1mM), lactate (10 mM), oligomycin (1 µM) and rotenone/antimycin A (0.5 µM). The data are expressed as the mean ± SEM (*n* = 4–6). **p < 0.01 ****p < 0.0001, compared with control; **p < 0.01 ****p < 0.0001 compared with lactate.

According to previous research, podocytes, like many other cell types, are capable of generating ATP through both glycolysis and oxidative phosphorylation^10^. In the first step of our study, we demonstrated that blocking MCT1 leads to lower insulin-dependent glucose utilization, which may be associated with impairments in glycolytic activity. To further investigate this, we measured the ECAR (Figs. 3A and 4A) and OCR (Figs. 3B and 4B) to determine the relative contribution of glycolysis and mitochondrial respiration to total ATP production. MCT1 inhibition significantly decreased total ATP production compared with control conditions (*p* < 0.05; Figs. 3C and 4C). Neither insulin nor lactate was able to restore ATP production under these conditions (*p* < 0.05; Figs. 3C and 4C). With regard to the proportion of glycolysis and mitochondrial respiration, insulin or lactate alone did not significantly affect the ATP production rate under basal conditions (Figs. 3D and 4D). In contrast, incubation of podocytes with the MCT1 inhibitor reduced the proportion of ATP derived from glycolysis (*p* < 0.05; Figs. 3D and 4D), indicating a shift toward mitochondrial respiration as the dominant energy source. A similar pattern was observed when insulin or lactate was combined with MCT1 inhibition, where ATP production remained predominantly OXPHOS-dependent (*p* < 0.001; Figs. 3D and 4D). This further confirms reliance on mitochondrial metabolism under MCT1-inhibited conditions.

### Effect of MCT1 Inhibition on the regulation of the glomerular filtration barrier and remodeling of the podocyte F-actin cytoskeleton

**Fig. 5:**
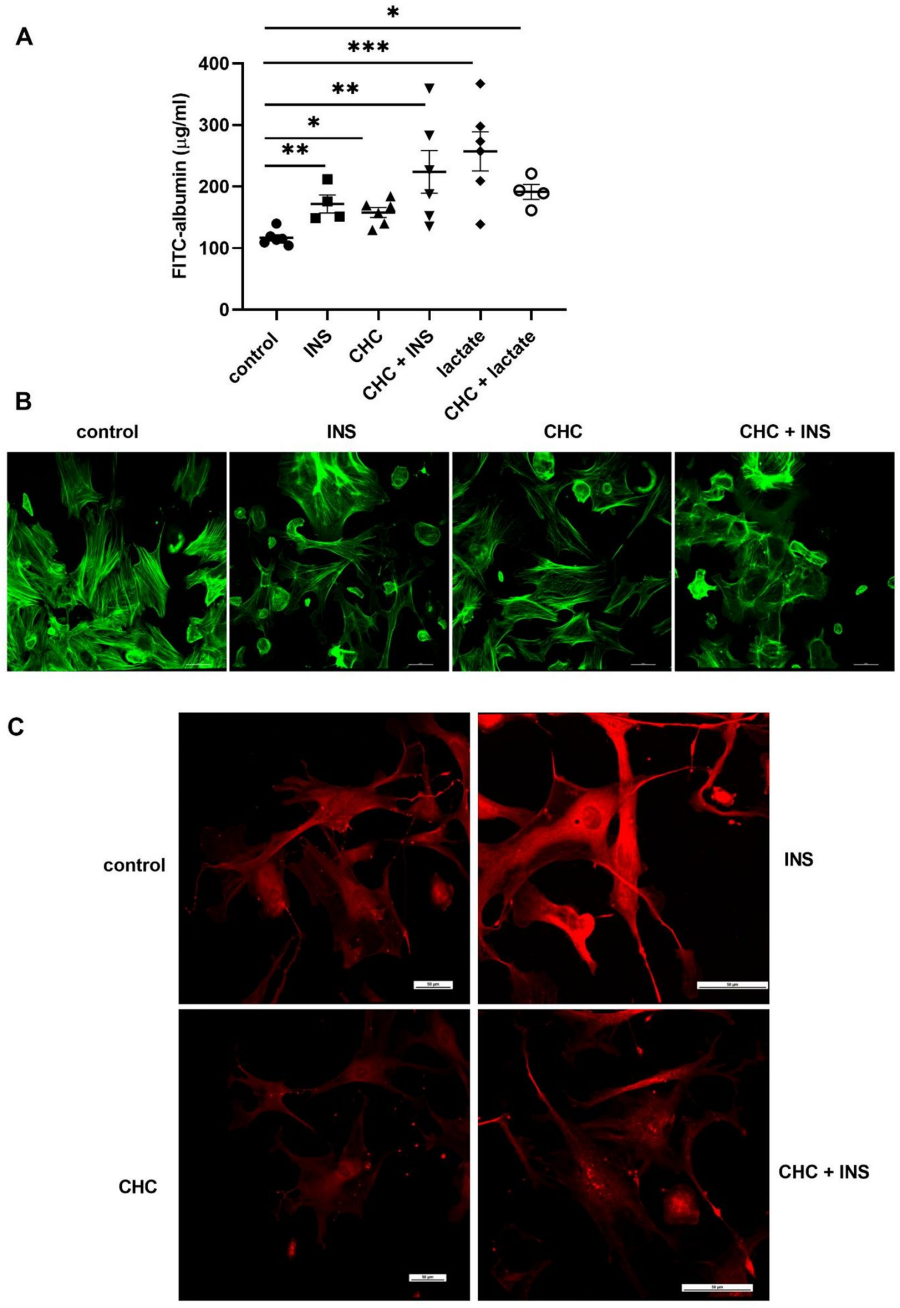
Effect of MCT1 inhibition on permeability to albumin across the podocyte monolayer and insulin-stimulated remodeling of the F-actin cytoskeleton. (**A**) Transepithelial permeability to albumin was evaluated by measuring the diffusion of FITC-labeled BSA across podocyte monolayers. The data are expressed as the mean ± SEM (*n* = 4–6). *p < 0.05, **p < 0.05, compared with control. (**B**) The F-actin network was labeled with isothiocyanate phalloidin and visualized with fluorescence microscopy. (**C**) Representative images of nephrin localization within podocytes. Cells were immunostained with anti-nephrin antibody.

**Fig. 6:**
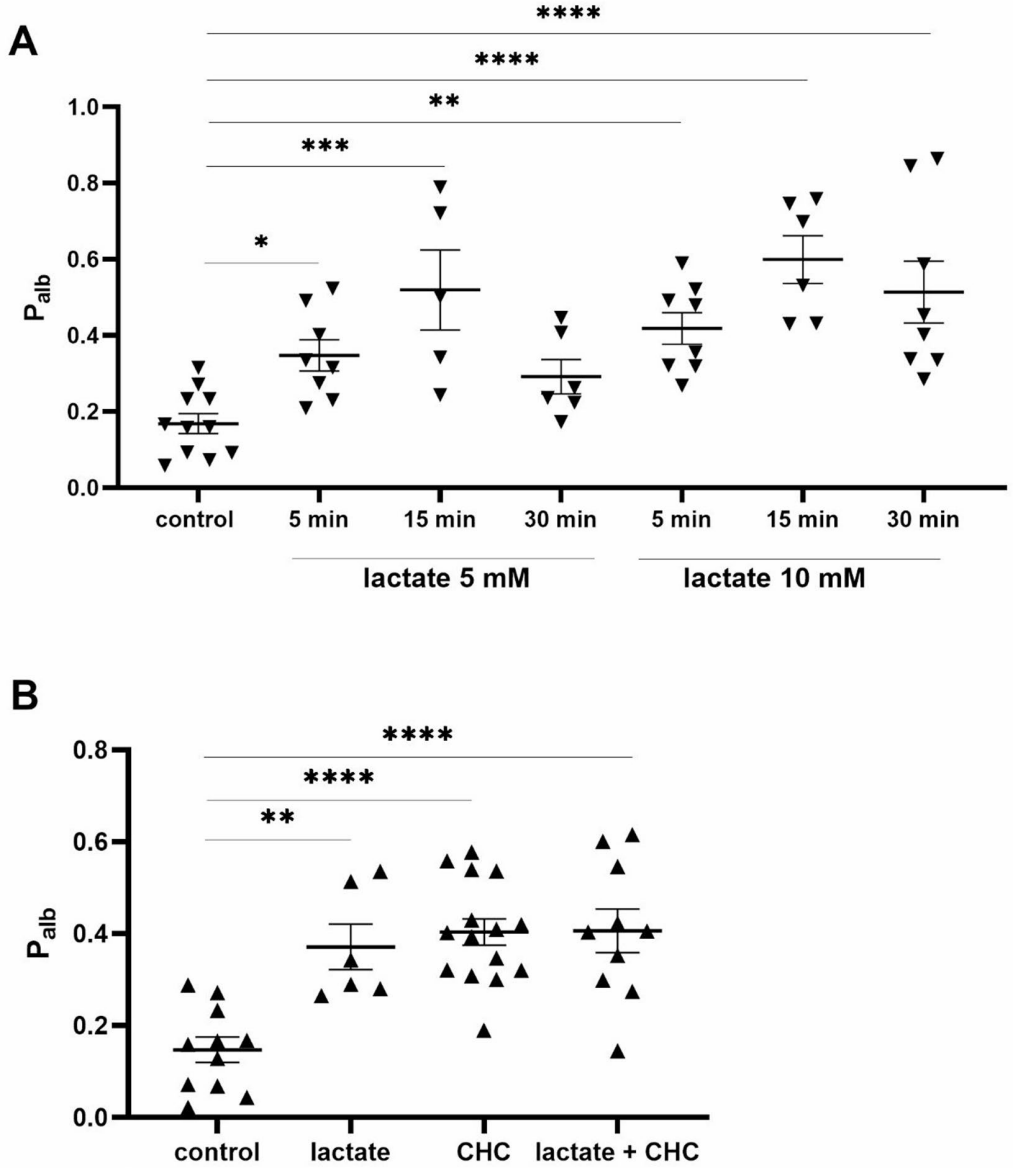
Effects of lactate on albumin permeability in isolated rat glomeruli (**A**). Glomerular permeability was evaluated at 5, 15, and 30 min following exposure to 5 or 10 mM lactate. The data are expressed as the mean ± SEM (*n* = 5–11). *p < 0.05 **p < 0.01 ***p < 0.001 ****p < 0.0001, compared with control. Effects of MCT1 inhibition on albumin permeability in isolated rat glomeruli in the presence of lactate (**B**). Glomerular permeability was assessed 5 min after exposure to 10 mM lactate, following 20 min of preincubation with 1 mM CHC. The data are expressed as the mean ± SEM (*n* = 6–15). **p < 0.01 ****p < 0.0001, compared with control.

Recent studies have shown that insulin can dynamically remodel the actin cytoskeleton in podocytes, contributing to retraction of their foot processes^38,39^. In our previous work, we observed increased GLUT4 and F-actin staining near the cell surface under glucose restriction conditions, which returned to baseline after lactate supplementation^16^. Based on these findings, we hypothesize that lactate metabolism plays a critical role in insulin-regulated actin cytoskeleton dynamics and glomerular permeability. To test this, we measured albumin flux across a podocyte monolayer. Both MCT1 inhibition and insulin increased podocyte permeability (*p* < 0.05, Fig. 5A). Interestingly, lactate alone induced more than a twofold increase in permeability (*p* < 0.001; Fig. 5A). Moreover, combined application of the MCT1 inhibitor with insulin or lactate further sustained the increase in podocyte permeability. (*p* < 0.05; Fig. 5A). Moreover, quantitative analysis revealed intracellular F-actin remodeling under the analyzed conditions (Fig. 5B). Both insulin and CHC treatments alone enhanced the formation of actin stress fibers near the cell surface compared with control cells, and combined treatment resulted in an even more pronounced effect (Fig. 5B). The immunofluorescence experiments showed substantial changes in the subcellular localization of nephrin, which is critical for stabilizing podocyte cytoarchitecture and the action of insulin^41,42^. Insulin treatment increased the amount of nephrin that was detected close to the cell surface and in the cytoplasm, and the MCT1 inhibitor significantly reduced the amount of protein compared with controls (Fig. 5C). Insulin administration did not reverse the effect of the MCT1 inhibitor on nephrin expression levels, but it altered the staining pattern from a homogeneous to a more granular distribution (Fig. 5C). These results confirmed that lactate regulates podocyte permeability and promotes actin fiber rearrangement. To further investigate, we next assessed the effect of lactate alone on the permeability of isolated glomeruli. (Fig. 6). Treatment with 5 mM or 10 mM lactate rapidly increased albumin permeability within 5 min, and this effect was sustained over time (Fig. 6A, *p* < 0.05). Therefore, in the following experiments we applied a 5-minute lactate exposure in the presence of CHC. MCT1 inhibition caused an increase in permeability similar to that induced by lactate alone, and their combined application produced no further effect (Fig. 6B, *p* < 0.01).

## Discussion

Podocytes primarily rely on glycolysis to meet their energy demands, likely because of the absence of mitochondria in their foot processes^20,21^. The inhibition of glycolysis has been shown to impair podocyte function by reducing lamellipodia length, compromising migratory capacity, and ultimately inducing apoptosis^20^. A key early adaptation in cells that are committed to a glycolytic phenotype is the upregulation of GLUTs and MCTs, which facilitate efficient substrate uptake^42,43^. In the present study, we found that MCT1 inhibition reduced insulin-stimulated glucose uptake and promoted a metabolic shift toward OXPHOS at the expense of glycolysis. Based on these findings, we hypothesize that the normal function of MCT1 is crucial for maintaining glucose metabolism and ensuring proper metabolic balance in podocytes. Similar findings were reported in adipocytes, in which lactate transport inhibition via MCT1 significantly reduced glycolytic flux in these cells^44^.

In this study, parallel to the reduction of glycolysis and insulin-dependent glucose uptake, MCT1 inhibition also affected the insulin signaling pathway. Specifically, it reduced insulin-stimulated Akt phosphorylation without altering IR phosphorylation. These results indicate that MCT1 is involved in insulin signaling within podocytes, likely functioning downstream of the IR, which requires further investigation. A similar dependence was previously observed in podocytes under hyperglycemic conditions. In that study, high glucose concentrations reduced the insulin effect on Akt phosphorylation in podocytes, while not affecting IR phosphorylation^46^. In present study, MCT1 inhibition also altered GLUT4 distribution. Podocytes rely on insulin-evoked GLUT4 translocation for glucose uptake, which we previously demonstrated as increased GLUT4 in membrane fractions following insulin stimulation^13^.. However, quantifying its plasma-membrane fraction is needed to confirm the effects of MCT1 inhibition on GLUT4 translocation.

Efficient lactate exchange via MCT helps maintain the balance between intracellular and extracellular lactate levels, thereby influencing the NADH/NAD⁺ ratio through an LDH-catalyzed reaction, which in turn regulates glycolytic activity. The inhibition of MCT1, a key lactate transporter, may have profound implications for lactate metabolism and consequently glucose homeostasis, potentially contributing to the development of metabolism-related disorders. Data from the literature highlight the therapeutic potential of MCT1 across a range of disease conditions. For example, the homozygous deletion of MCT1 is embryonically lethal in mice^46^. In neurodegenerative disorders, restoring MCT1 expression and lactate homeostasis significantly promotes axonal regeneration and improves functional recovery^48^. Moreover, during ischemia, MCT1-dependent lactate uptake supports macrophage polarization toward an M2-like phenotype, promoting revascularization and muscle regeneration^49^. Conversely, MCT1 is frequently overexpressed in cancer cells^51^, and its inhibition has shown promise in improving the efficacy of anti-cancer therapies^52,53^ by blocking lactate utilization as an alternative energy source, thereby suppressing tumor growth. Thus, MCT1 serves as a critical regulator of cellular metabolism, positioning it as a key player in the pathophysiology of metabolic diseases.

We propose an emerging contribution of lactate metabolism and MCT1-mediated transport to renal function. Our previous study demonstrated that hyperglycemia reduces MCT1 protein levels and LDH activity^55^, leading to impairments in glycolytic flux, the disruption of intracellular glucose transport, and the loss of podocyte foot processes. These changes were accompanied by alterations of the expression and localization of key proteins for insulin signaling, such as GLUT4 and nephrin^55^. Based on these findings, we hypothesize that hyperglycemia-induced alterations of podocyte morphology and glucose metabolism may result from disruptions of the lactate transport system. This hypothesis is supported by the present findings, in which MCT1 inhibition modified the response of podocytes to insulin and decreased glucose uptake. Furthermore, this disruption triggered actin cytoskeleton reorganization, altered key proteins (e.g., nephrin), and increased permeability of the podocyte layer. Our study demonstrated that MCT1 inhibition, as well as exposure to lactate or insulin, increased glomerular permeability. Given that actin dynamics are controlled by multiple pathways, these changes likely reflect the activation of distinct signaling mechanisms. Insulin promotes actin remodeling through the canonical insulin–Akt–GLUT4 pathway^56^, whereas lactate may act via alternative mechanisms that modulate actin organization. One possible mechanism involves the cAMP–PKA axis. Lactate can lower intracellular cAMP and suppress PKA activation through GPR81^58^, whereas elevated cAMP in endothelial cells is generally associated with reduced permeability^58^. Notably, both lactate exposure and MCT1 inhibition produced similar effects, which may be explained by the accumulation of extracellular lactate when its cellular uptake is blocked.

The literature suggests that impairments in MCT1 function contribute to the development of insulin resistance in various cells and tissues. In mice, MCT1 deficiency in adipose tissue exacerbates high-fat diet-induced insulin resistance by promoting inflammation^23^. Additionally, hepatic deletion of the gene that encodes MCT1 alters the expression of numerous genes that are involved in lipid synthesis, leading to greater lipid accumulation in the liver and promoting the development of high-fat diet-induced obesity^61^. Elevations of plasma lactate levels are commonly observed in diabetes, and chronic extracellular lactic acidosis is acknowledged as a negative prognostic marker for the disease^60^. The mechanism that underlies this phenomenon may involve the inhibition of MCT1, which impairs cellular lactate uptake and utilization, leading to an increase in plasma lactate concentration. This is supported by findings in obese Zucker rats, in which hyperlactatemia is associated with lower MCT1 and MCT4 expression in skeletal muscle and lower lactate uptake^61^. Similarly, in patients with type 2 diabetes, MCT1 content is reduced in skeletal muscle^62^. Moreover, Choi et al^65^. proposed that an elevation of plasma lactate levels suppressed insulin-stimulated glycolysis, an effect that may precede the development of insulin resistance. Podocytes are insulin-sensitive cells and particularly susceptible to insulin resistance, and disruptions of lactate metabolism appear to contribute to the development of this condition. These data provide valuable insights into the clinical assessment of kidney damage. Insulin resistance can be detected early, even when the glomerular filtration rate remains normal, and it becomes nearly universal as chronic kidney disease progresses, particularly in end-stage kidney failure^64,65^. Moreover, podocyte injury is not only an early event in the development of glomerular diseases but also a critical factor in the worsening of kidney function. Therefore, understanding the precise mechanisms of the development of insulin resistance at an early stage could greatly enhance the diagnosis and treatment of such pathological states.

Although MCT1 inhibition can negatively impact energy metabolism, several studies have also reported beneficial effects. For example, *Slc16a1* deletion in the intestinal epithelium in male mice was protective by improving systemic glucose homeostasis, lowering circulating levels of proinflammatory cytokines (i.e., tumor necrosis factor α and C-C motif ligand 2), and attenuating the inflammatory response in intestinal macrophages^66^. These effects were attributed to the blockade of the intestinal absorption of lactate and short-chain fatty acids and were partially dependent on alterations of the composition of the gut microbiota. MCT1 in the intestinal epithelium plays a crucial role in the absorption of short-chain fatty acids, which are generated by the microbial fermentation of dietary fiber. Short-chain fatty acids serve as primary nutritional sources for colonic epithelial cells, supporting intestinal barrier integrity and modulating immune responses^67^. Thus, lactate transport via MCT1 appears to regulate intestinal macrophage activity and local inflammation, which can subsequently influence systemic inflammation and impairs glucose tolerance and insulin sensitivity. Notably, MCT function, including the direction of lactate exchange and substrate specificity, is tissue-specific and governed by electrochemical gradients of monocarboxylates and protons^68,69^.

Based on our previous findings that MCT1 protein levels decreased in response to extracellular lactate, we hypothesized that MCT1 mediates lactate uptake in podocytes^16^. This downregulation may reflect a reduction of the cellular demand for lactate in the context of its extracellular accumulation. In the present study, MCT1 inhibition was expected to reduce intracellular lactate availability, thereby limiting the substrate supply for oxidative metabolism. Our findings suggest that MCT1 inhibition and the resulting blockade of lactate uptake disrupt critical energy pathway by suppressing glycolysis and promoting a shift toward OXPHOS. This metabolic reprogramming may represent an adaptive response to energy stress, potentially associated with an increase in mitochondrial biogenesis. This is particularly notable when considering that MCT1 inhibition led to an overall reduction of the total cellular ATP pool. Notably, MCT1 inhibition reduced the total cellular ATP pool, and even in the presence of insulin or lactate this compensatory shift failed to restore ATP levels to basal conditions. Given that an increase in the NAD⁺/NADH ratio is considered a key driver of mitochondrial biogenesis^70^, these observations support the notion that impairments in lactate metabolism alter redox balance in favor of NAD⁺ accumulation, thereby promoting mitochondrial adaptation. Mitochondria are highly dynamic organelles that are able to undergo continuous biogenesis, fissions, and fusions, thereby allowing remodeling of the mitochondrial network in response to metabolic demands or stress and ensuring efficient energy production and distribution^71^. Although these conclusions are supported by bioenergetic analysis indicating a shift toward OXPHOS, they warrant further investigation of proteins governing mitochondrial biogenesis and dynamics. However, our hypothesis is further supported by Zhang et al^72^., who reported that MCT1 deletion in skeletal muscle led to a significant increase in mitochondrial content, the upregulation of mitochondrial respiratory chain subunits, and an increase in the expression of peroxisome proliferator-activated receptor γ coactivator 1α (PGC-1α), a master regulator of mitochondrial biogenesis. These changes were accompanied by an increase in the OCR, indicating a shift toward a more oxidative metabolic phenotype. An increase in NAD⁺ levels that resulted from MCT1 loss and lower mitochondrial LDH activity contributed to sirtuin 1 activation, which in turn deacetylates and activates PGC-1α. These findings align with our earlier work, which demonstrated that an elevation of extracellular lactate, followed by the suppression of glycolytic flux, triggered an increase in OXPHOS activity^21^. In parallel, lactate exposure promoted mitochondrial biogenesis, evidenced by an increased mitochondrial DNA content and the upregulation of biogenesis markers such as PGC1α and TFAM^21^. Interestingly, under physiological conditions, the podocyte-specific knockout of genes that are essential for mitochondrial biogenesis and dynamics (e.g., *PGC-1α*, *Tfam*, and *DRP1*) did not result in glomerular or renal injury in mice^17^. This suggests that glycolysis alone may be sufficient to meet baseline energy requirements of podocytes. However, in a pathological context, *PGC-1α* overexpression and the resulting increase in mitochondrial biogenesis appeared to exert protective effects, as shown in models of adriamycin-^73^ and aldosterone-induced^8^ podocyte injury. Enhancing mitochondrial respiration under pathological conditions not only helps restore energy homeostasis by increasing ATP production but also reestablishes redox balance by normalizing the NAD⁺/NADH ratio. These findings highlight the importance of the intracellular lactate shuttle in maintaining cellular redox status, emphasizing its role in regulating NAD⁺/NADH balance.

**Fig. 7:**
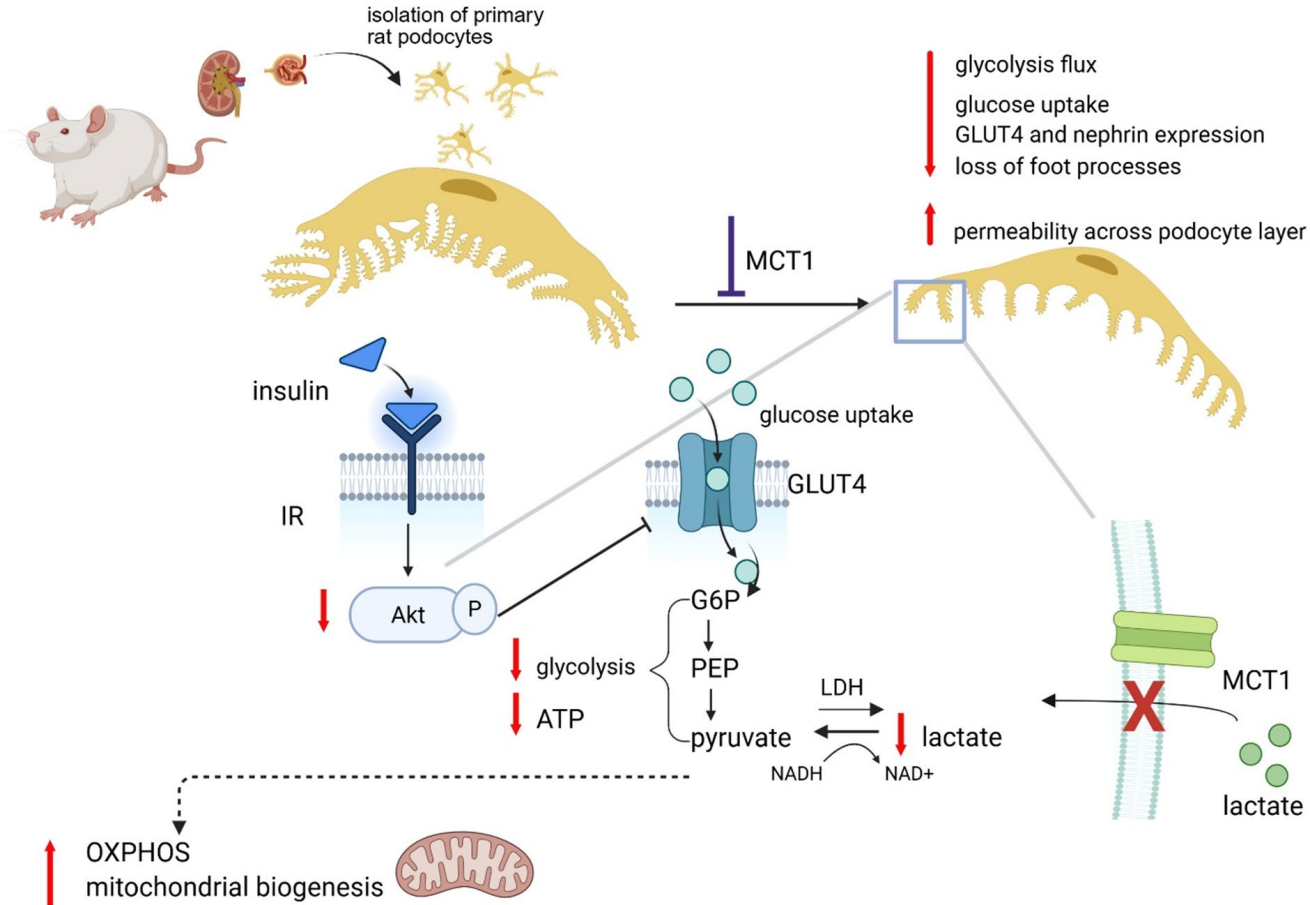
Proposed effects of MCT1 inhibition on podocyte energy metabolism and function. Created with BioRender.com (accessed September 2025, https://www.biorender.com).

In summary, the regulation of intracellular lactate levels via MCT1 is crucial for maintaining metabolic balance in podocytes. These cells primarily depend on glycolysis for energy, and disruptions of lactate transport, which is essential for sustaining glycolytic flux, can impair glucose metabolism, insulin signaling, and cytoskeletal integrity and contribute to podocyte dysfunction as demonstrated in our study by increasing albumin permeability. Enhancing mitochondrial respiration may represent a compensatory response, helping to meet high energy demands and preserving podocyte function under stress (Fig. 7).

## Supplementary Information

Below is the link to the electronic supplementary material.

Supplementary Material 1

## Data Availability

The datasets generated during the current study are not publicly available but are available from the corresponding author on reasonable request.

## Abbreviations

ATP: adenosine triphosphate
BSA: bovine serum albumin
CHC: α-cyano-4-hydroxycinnamic acid
ECAR: extracellular acidification rate
GLUT4: glucose transporter 4
LDH: lactate dehydrogenase
MCT: monocarboxylate transporter 1
NAD: nicotinamide adenine dinucleotide
OCR: oxygen consumption rate
OXPHOS: oxidative phosphorylation
PBS: phosphate-buffered saline
PGC-1α: peroxisome proliferator-activated receptor γ coactivator 1α
SCFA: short-chain fatty acid
SFM: serum-free RPMI1640
