# Supplementary figures and images for: MCT1 as a critical regulator of insulin signaling, energy homeostasis and podocyte function
Source: Sci Rep. 2026 Jan 21;16:5906. doi: 10.1038/s41598-026-37093-x (PMC12894881; doi:10.1038/s41598-026-37093-x)

**Akt**

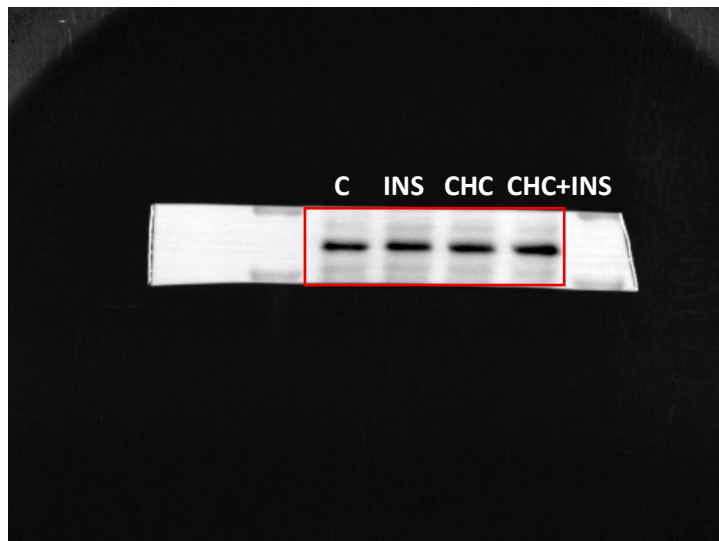

**p-Akt**

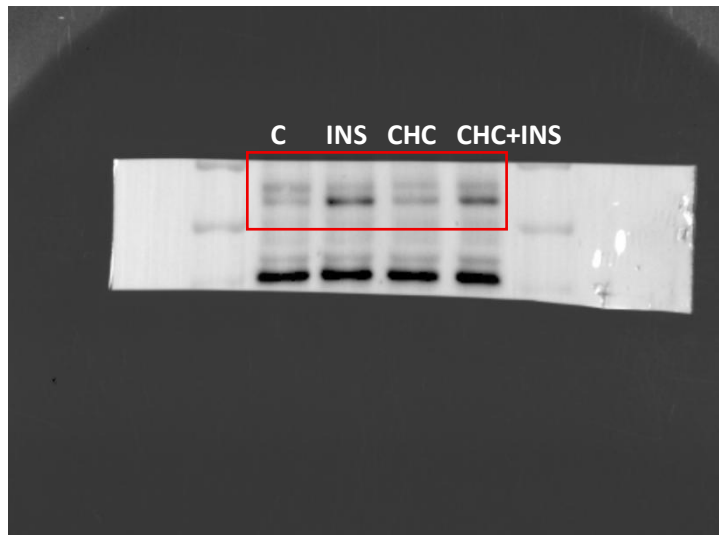

**actin**

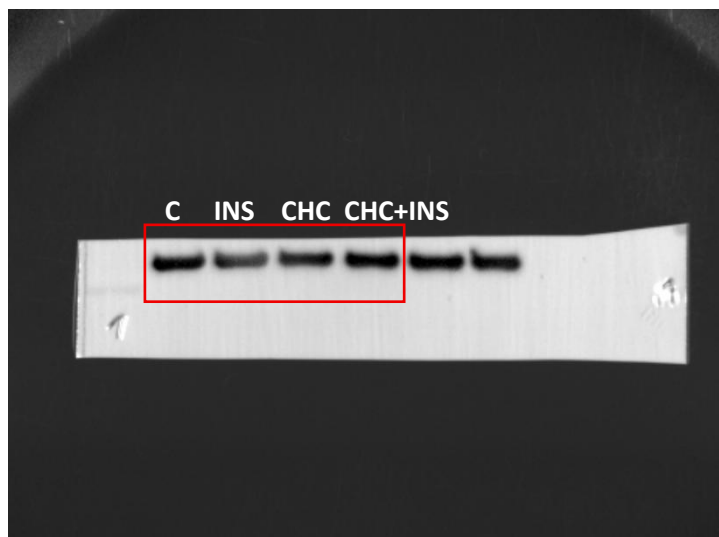

## MCT1

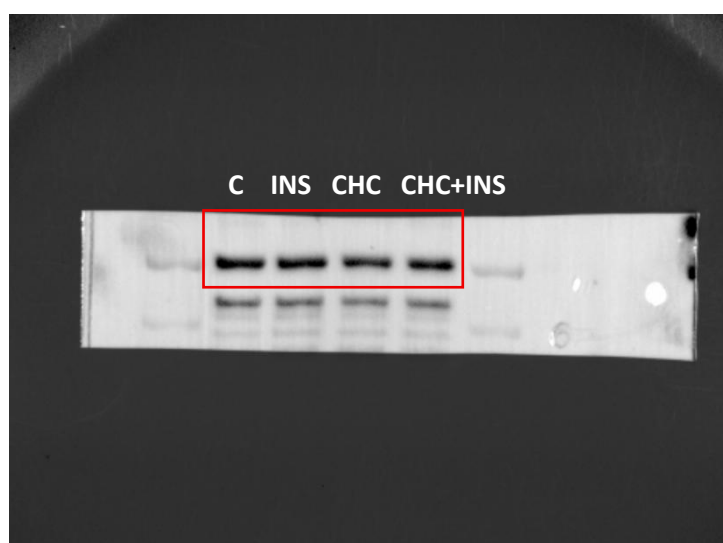

## actin

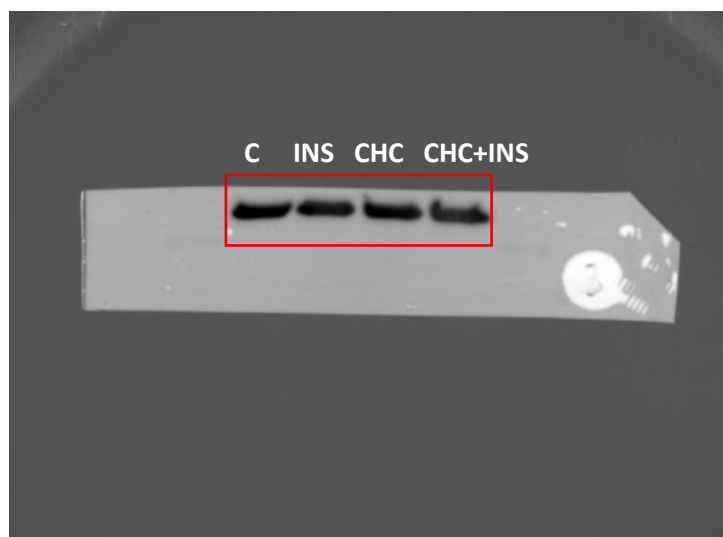

IR

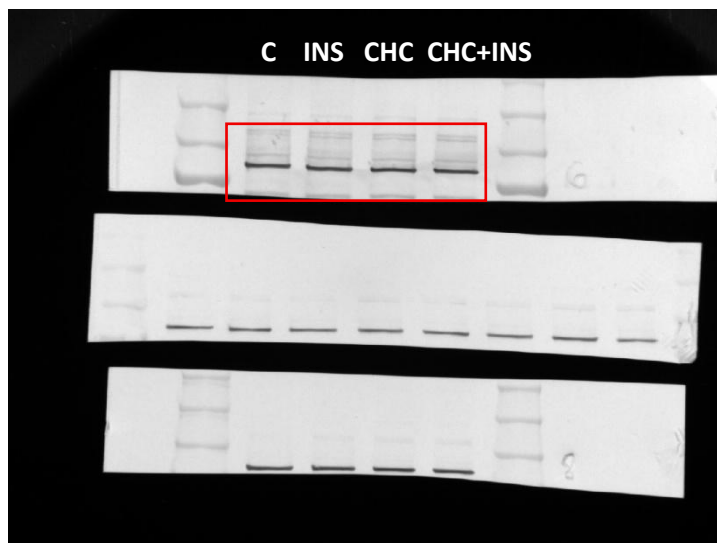

p-IR

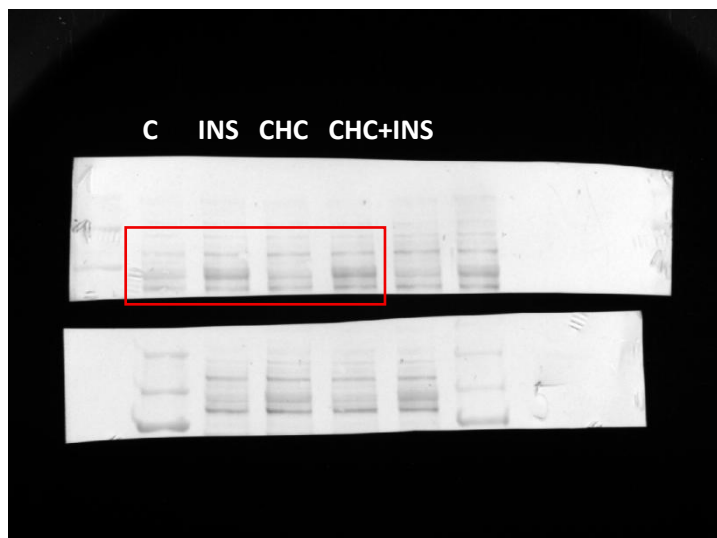

actin

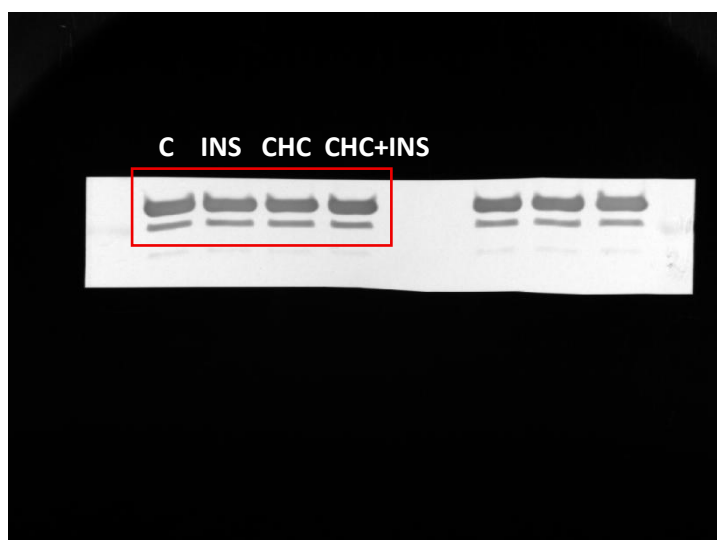

Supplement: Supplementary file 1 — Supplementary Material 1 [file 41598_2026_37093_MOESM1_ESM.pdf]
